# Corylin Suppresses Hepatocellular Carcinoma Progression via the Inhibition of Epithelial-Mesenchymal Transition, Mediated by Long Noncoding RNA GAS5

**DOI:** 10.3390/ijms19020380

**Published:** 2018-01-27

**Authors:** Chi-Yuan Chen, Chin-Chuan Chen, Tzong-Ming Shieh, Chuen Hsueh, Shu-Huei Wang, Yann-Lii Leu, Jang-Hau Lian, Tong-Hong Wang

**Affiliations:** 1 Tissue Bank, Chang Gung Memorial Hospital, Tao-Yuan 33305, Taiwan; d49417002@gmail.com (C.-Y.C.); chinchuan@mail.cgu.edu.tw (C.-C.C.); ch9211@cgmh.org.tw (C.H.); 2 Graduate Institute of Health Industry Technology and Research Center for Industry of Human Ecology, College of Human Ecology, Chang Gung University of Science and Technology, Tao-Yuan 33303, Taiwan; 3 Graduate Institute of Natural Products, Chang Gung University, Tao-Yuan 33303, Taiwan; ylleu@mail.cgu.edu.tw; 4 Department of Dental Hygiene, College of Health Care, China Medical University, Taichung 40402, Taiwan; tmshieh@mail.cmu.edu.tw; 5 Department of Anatomic Pathology, Chang Gung Memorial Hospital, Chang Gung University School of Medicine, Tao-Yuan 33305, Taiwan; 6 Department of Anatomy and Cell Biology, College of Medicine, National Taiwan University, Taipei 10617, Taiwan; shwang@ntu.edu.tw; 7 Chinese Herbal Medicine Research Team, Healthy Aging Research Center, Chang Gung University, Tao-Yuan 33303, Taiwan; 8 Center for Traditional Chinese Medicine, Chang Gung Memorial Hospital, Tao-Yuan 33305, Taiwan; 9 Genomic Medicine Core Laboratory, Chang Gung Memorial Hospital, Tao-Yuan 33305, Taiwan; a24255544@gmail.com; 10 Liver Research Center, Department of Hepato-Gastroenterology, Chang Gung Memorial Hospital, Tao-Yuan 33305, Taiwan

**Keywords:** corylin, hepatocellular carcinoma, epithelial-mesenchymal transition, lncRNA GAS5

## Abstract

Corylin is a flavonoid extracted from the nuts of *Psoralea corylifolia* L. (Fabaceae), which is a widely used anti-inflammatory and anticancer herb in China. Recent studies revealed antioxidant, anti-inflammatory, and bone differentiation–promoting effects of corylin. However, there are no studies examining the anticancer activity of corylin. In this study, we used cells and animal models to examine the antitumor effects of corylin on hepatocellular carcinoma (HCC) and then studied its downstream regulatory mechanisms. The results showed that corylin significantly inhibited the proliferation, migration, and invasiveness of HCC cells and suppressed epithelial–mesenchymal transition. We found that the anti-HCC mechanism of corylin’s action lies in the upregulation of tumor suppressor long noncoding RNA growth arrest-specific transcript 5 (GAS5) and the activation of its downstream anticancer pathways. In animal experiments, we also found that corylin can significantly inhibit tumor growth without significant physiological toxicity. The above results suggest that corylin has anti-HCC effects and good potential as a clinical treatment.

## 1. Introduction

Hepatocellular carcinoma (HCC) is ranked sixth in incidence among cancers worldwide [1,2]. In Taiwan, HCC is the second leading cause of death due to cancer [3]. Currently, the mainstay treatment of HCC is surgical resection. When HCC progresses to a late stage and cannot be resected, chemotherapy is the only therapeutic option [4,5]. Nevertheless, HCC can easily metastasize and it has primary multidrug resistance, which results in poor efficacy of chemotherapeutic agents [6,7,8]. In addition, most chemotherapeutic agents have strong adverse effects, which severely affect the patient’s quality of life [9,10,11]. Therefore, the development of effective treatment methods with milder adverse effects has always been a focus of HCC research.

The use of traditional Chinese medicine (TCM) in the treatment of diseases has a long history in China, and TCM research has gradually gained international recognition in recent years [12,13,14,15]. Compared with Western medicine, TCM provides effective treatment options with relatively mild adverse effects [16]. Nonetheless, differences in the quality of TCM therapeutics and in the concentrations of their endogenous, biologically active ingredients usually result in variable therapeutic efficacy and limit their clinical applications [17]. Therefore, to enhance and stabilize the therapeutic efficacy of TCM, many studies have been conducted to identify and purify their biologically active ingredients [18,19,20,21]. With developments in separation technologies (such as high-performance liquid chromatography (HPLC), capillary electrophoresis (CE), gas chromatography (GC), and thin-layer chromatography (TLC)) and mass spectrometry, the active ingredients of many TCM therapeutics have been successfully purified and their functions determined. The compounds identified can be used at lower doses and have more specific therapeutic efficacy. Currently, many compounds extracted from TCM therapeutics, e.g., artemisinin and curcumin, are used in the treatment of cancer and have shown good efficacy [22,23,24,25,26]. Some of these drugs have been demonstrated to significantly inhibit the proliferation and metastasis of HCC cells and can significantly increase the survival period of patients [27,28,29,30].

*Psoralea corylifolia* L. (Fabaceae) is a TCM herb that is popular in many Asian countries for the treatment of bacterial infections, inflammation, and some cancers [31,32,33,34]. *P. corylifolia* L. contains polyphenols, such as psoralen, isopsoralen, and psoralidin; flavonoids, such as bavachin, isobavachalcone, and neobavaisoflavone; and the aromatic compound bakuchiol, which has been found to have biological activities and various therapeutic effects [32]. Corylin is a flavonoid that is extracted from the nuts of *P. corylifolia* L. Studies have revealed that corylin can promote bone differentiation and bone growth and prevent osteoporosis [35,36]. In addition, recent studies indicate that corylin has anti-inflammatory effects that can inhibit inflammatory responses induced during a bacterial infection by suppressing inducible NO synthase (iNOS) and cyclooxygenase (COX) expression [33,37]. However, thus far, there have been no studies on its anticancer effects. In this study, we investigated the anticancer activity of corylin and then studied its target regulatory mechanisms.

In the past, studies on effector mechanisms of drugs have mostly focused on the examination of the effects at the protein level. In contrast, recent studies revealed that noncoding RNAs (such as microRNAs and long noncoding RNAs (lncRNAs)) also play important roles in the physiological regulation of cellular functions [38,39]. Among noncoding RNAs, lncRNAs are longer than 200 nt and account for 60% of the human genome. Studies have shown that lncRNAs can participate in (and regulate) many cellular and physiological processes, such as chromosomal modifications, transcription, translation, and protein activation [40,41,42,43]. It has been demonstrated that many drugs can regulate lncRNAs as a mechanism of therapeutic efficacy in terms of anticancer pathways [44,45]. In this study, we found that corylin can induce anticancer lncRNA growth arrest-specific transcript 5 (GAS5) and inhibit epithelial–mesenchymal transition (EMT), thereby inhibiting the proliferation, migration, and invasiveness of HCC cells. The results of animal experiments also confirmed that corylin can significantly inhibit the growth of tumors in mice and does not exert significant toxicity in mice. These data show that corylin has possible applications for HCC treatment.

## 2. Results

### 2.1. Corylin Inhibits the Proliferation, Migration, and Invasiveness of HCC Cells

**Figure 1:**
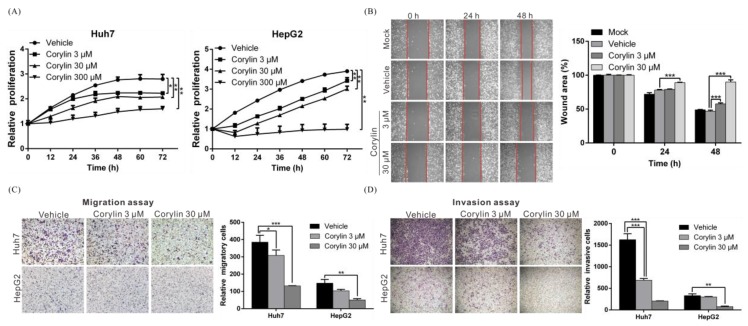
Corylin inhibits the proliferation, migration, and invasiveness of hepatocellular carcinoma (HCC) cells. (**A**) The cell proliferation capacities of Huh7 and HepG2 were monitored at the indicated time points using an xCELLigence real-time cell analyzer. Corylin significantly inhibited the proliferative capacities of both cell lines. *p* < 0.05 (*), *p* < 0.01 (**). Data are expressed as the mean ± S.D. of three independent experiments; (**B**) Wound-healing abilities were compared between corylin- and vehicle-treated Huh7 cells (left panel). Corylin reduced the wound-healing ability of Huh7 cells. The quantitative wound-healing assay results are shown in the right panel. *p* < 0.001 (***). Magnification: 100×; (**C**) Cell migration capacity was compared between Huh7 and HepG2 cells treated with/without corylin using a Transwell assay (left panel). Corylin significantly reduced cell migratory ability in both cell lines. Quantitative cell migration assay results are shown in the right panel; (**D**) Invasion assays were performed using Matrigel-coated polyethylene terephthalate membrane inserts. Corylin significantly inhibited cell invasion ability in both cell lines at a concentration of 30 μM (left panel). Quantitative cell invasion assay results are shown in the right panel. All experiments were performed in triplicate. Mock: cells treated with Dulbecco’s modified Eagle medium (DMEM). Vehicle: cells treated with dimethyl sulfoxide (DMSO). *p* < 0.05 (*), *p* < 0.01 (**), *p* < 0.001 (***). Magnification: 100×.

To understand whether corylin exerts anticancer action on HCC cell lines, we used different concentrations of corylin (3, 30, or 300 μM) to treat HCC cell lines HepG2 and Huh7 and employed the xCELLigence real-time cell analyzer (ACEA Biosciences, Inc., San Diego, CA, USA) to study the effects of corylin on cell proliferation. We found that compared with the no-treatment control group, as little as 3 μM corylin inhibited the proliferation of both cell lines in a dose-dependent manner. The half-maximal inhibitory concentration (IC_50_) of corylin toward Huh7 and HepG2 cells was calculated using GraphPad Prism software (Version 6, GraphPad Software, Inc., San Diego, CA, USA) and was 30 and 10 μM, respectively. The results showed that at the corylin concentration of 30 μM, the proliferation of HepG2 and Huh7 cells was significantly inhibited by 37% and 24% at 48 h, respectively (Figure 1A).

Cancer cell invasion and metastasis are the main sources of cancer refractoriness. To further clarify whether corylin can inhibit the metastasis and invasiveness of HCC cells, we conducted a wound-healing assay and a Transwell migration assay to test whether corylin can affect cell migration. The results showed that 3 μM corylin significantly inhibited cell migration and inhibitory effects increased with the concentration of corylin (Figure 1B,C). At a concentration of 30 μM, corylin inhibited Huh7 and HepG2 migration by 66% and 67%, respectively (Figure 1C). Furthermore, corylin inhibited cell invasion, suppressing up to 87% of control cell invasion at a concentration of 30 μM (Figure 1D).

### 2.2. Corylin Inhibits Epithelial-Mesenchymal Transition (EMT)

**Figure 2:**
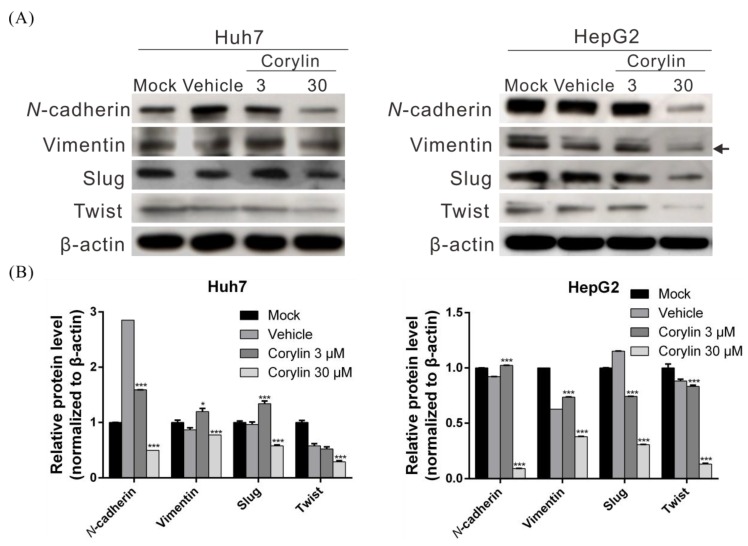
Corylin suppressed the migration and invasion capacities of HCC cell via inhibiting epithelial-mesenchymal transition (EMT). (**A**) The expression of EMT-related proteins in Huh7 and HepG2 cells after treatment with corylin or vehicle were analyzed by Western blotting. β-actin served as an internal control. A dose of 30 μM corylin significantly reduced the expression of EMT-related proteins in both cells. Densitometric analyses are shown in (**B**). The histogram shows the relative expression level of EMT-related proteins in the corylin-treatment compared to the mock-treatment group. Data are expressed as the mean ± S.D. of three independent experiments. * *p* < 0.05, *** *p* < 0.001, when compared to vehicle control. Mock: cells treated with DMEM medium. Vehicle: cells treated with DMSO.

EMT is an important process for cancer metastasis, weakening cell-cell adhesion and making cells more prone to migration. To determine whether corylin regulates EMT when inhibiting HCC migration and invasion, we carried out Western blotting to analyze the effects of corylin on EMT-associated proteins. The results indicated that the expression of EMT-promoting proteins such as N-cadherin, vimentin, slug, and twist significantly decreased in 30 μM corylin-treated cells compared with the control group (Figure 2A,B). These findings suggested that corylin can inhibit HCC migration and invasion by suppressing EMT.

### 2.3. Corylin Inhibits Tumor Growth in Mice

**Figure 3:**
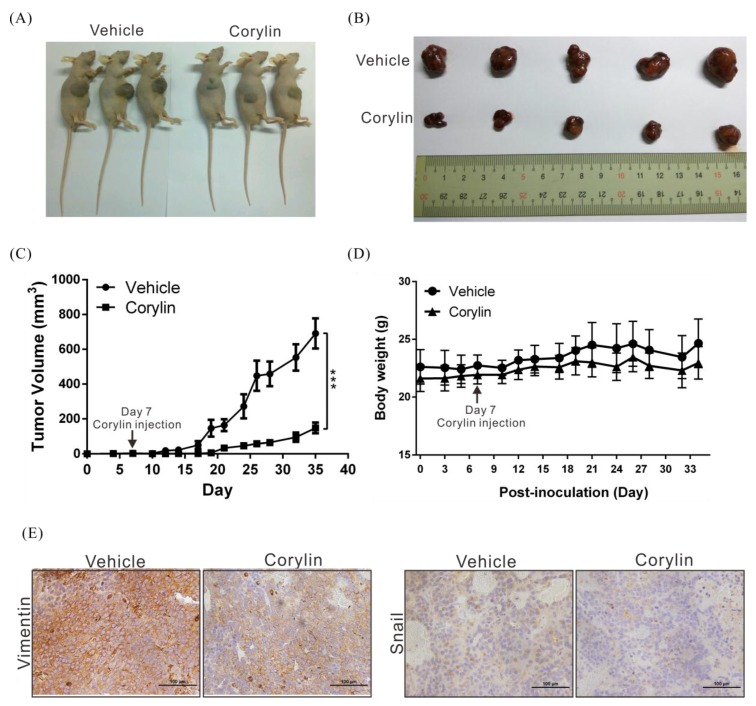
Corylin suppresses tumor growth in mice. (**A**,**B**) Huh7 cells (5 × 10^6^) were implanted into nude mice (*n* = 5). Representative images show the tumor xenografts at 35 days after implantation. Corylin (60 mg/kg, IP) significantly reduced tumor growth. IP: intraperitoneal injection; (**C**) Tumor volumes were measured every three days after implantation, and the volume of each tumor was calculated (length × width^2^ × 0.5). Bars indicate S.D. *** *p* < 0.001; (**D**) Body weights were calculated every three days after implantation. Mouse body weights in all groups did not significantly differ; (**E**) Immunohistochemical staining showed that corylin reduced EMT-related protein expression levels. Magnification: 400×. Scale bar = 100 μm.

To verify the anticancer effects of corylin, a mouse xenograft model was used to analyze the effects of corylin on tumor growth in mice. As in cell models, we found that corylin significantly inhibited tumor growth in vivo. Mice treated with corylin had tumors that were up to 87% smaller than those in the control group (vehicle only; Figure 3A–C). In addition, there were no significant differences in body weight between corylin-treated mice and control mice (Figure 3D), indicating that corylin may not have physiological toxicity at the dose tested. Immunohistochemical staining was performed to study the expression of EMT-associated proteins in mice. We found that corylin obviously downregulated EMT-promoting proteins such as vimentin and snail (Figure 3E). The results of this experiment are identical to those of the cellular experiments, suggesting again that corylin may inhibit EMT.

### 2.4. Corylin Can Inhibit the Activation of Signaling Pathways Associated with Cell Growth and Apoptosis

**Figure 4:**
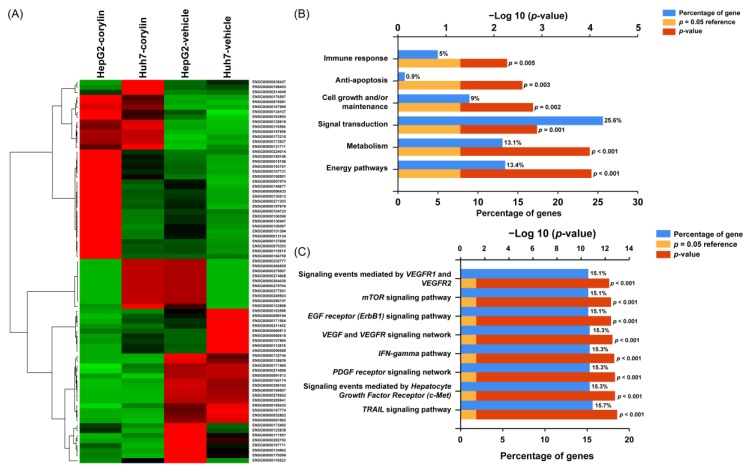
Corylin affected the expression of genes associated with cell growth and apoptosis pathways. (**A**) Heatmap comparing significant differentially expressed lncRNAs in Huh7 and HepG2 cells treated with or without corylin. Gene expression was significantly changed compared to that of the control group after corylin treatment. Bar charts represent the enriched biological processes (**B**) and biological pathways (**C**) associated with the differentially expressed genes after corylin treatment. Corylin affected the expression of genes associated with cell growth and apoptosis pathways.

To elucidate the anticancer mechanisms of action of corylin, we used doses of 30 μM and 10 μM to treat Huh7 and HepG2 cells, respectively, and carried out whole-transcriptome sequencing to identify the genes and signaling pathways that may be regulated by corylin. Heatmap analysis showed that after corylin treatment, gene expression was significantly changed compared with that of the control group (Figure 4A). We next employed Ingenuity Pathway Analysis and found that corylin affected the expression of genes associated with cell growth and apoptosis pathways, such as vascular endothelial growth factor (VEGF)-, mammalian target of rapamycin (mTOR)-, mesenchymal-epithelial transition factor (c-MET)-, tumor necrosis factor-related apoptosis-inducing ligand (TRAIL)-, and epidermal growth factor receptor (EGFR)-mediated signaling pathways (Figure 4B,C). The above results revealed that corylin may modulate cell growth and apoptosis pathways and thereby inhibit the proliferation and metastasis of HCC cells.

### 2.5. Corylin Exerts Its Anticancer Effects by Inducing lncRNA GAS5

**Figure 5:**
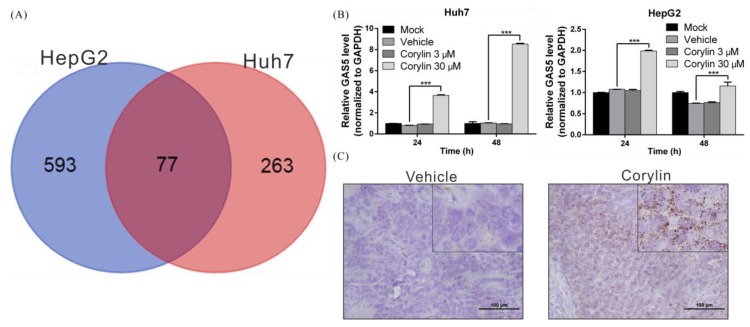
Corylin increases the expression of long noncoding RNA GAS5 in HCC cell lines. (**A**) The whole-transcriptome sequencing data revealed that 77 long noncoding RNAs (lncRNAs) manifested more than a 2-fold change in expression after corylin treatment in both cell lines; (**B**) Huh7 and HepG2 cells were treated with corylin for 48 h, and GAS5 expression was analyzed by quantitative real-time RT-PCR, which showed that 30 μM corylin significantly induced GAS5 expression. *** *p* < 0.001; (**C**) Representative results of the in situ hybridization of lncRNA GAS5 in the xenografts of mice treated with corylin or vehicle. Corylin significantly induced GAS5 expression. Scale bar = 100 μm. Error bars indicate S.D.

It is known that lncRNAs regulate many important physiological and drug responses [44,45]. To understand the role of lncRNAs in the anticancer mechanisms of action of corylin, we analyzed the abovementioned transcriptome sequencing data, and found that 77 lncRNAs manifested more than a 2-fold change in expression after corylin treatment when compared to the control group (Figure 5A). Among these lncRNAs, GAS5 was recently reported to be a tumor suppressor lncRNA because it has been shown to inhibit HCC proliferation and EMT progression by inhibiting the expression of genes such as vimentin [46]. To confirm the regulatory relation between corylin and GAS5, we carried out real-time PCR to determine the expression status of GAS5 in HCC cell lines. We found that corylin maximally upregulated GAS5 in Huh7 and HepG2 cells 7.2-fold and 1.8-fold, respectively (Figure 5B). We next used in situ hybridization to analyze the expression status of GAS5 in mouse tumor tissues and saw that GAS5 expression was notably higher in tumor tissues from corylin-treated mice in comparison with those from the control group (DMSO only; Figure 5C). These findings indicate that corylin may trigger GAS5-mediated anticancer mechanisms and may inhibit HCC progression.

**Figure 6:**
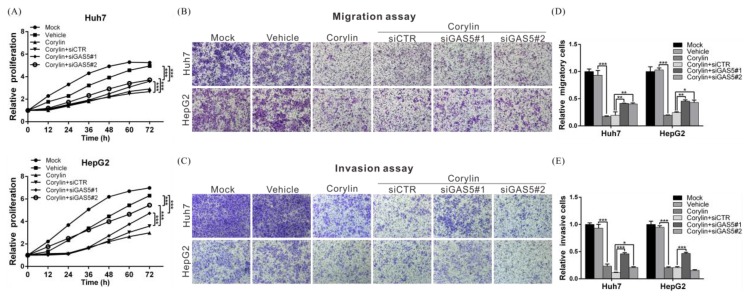
Corylin inhibits the proliferation, migration, and invasion of HCC cells by inducing lncRNA GAS5. (**A**–**C**) The inhibitory effects of corylin on cell proliferation, migration, and invasion were significantly reversed by treatment with GAS5 siRNA in the Huh7 and HepG2 cells. Magnification: 100×. Quantitative cell migration and invasion assay results are shown in (**D**,**E**). *p* < 0.05 (*), *p* < 0.01 (**), *p* < 0.001 (***). Error bars indicate S.D.

To confirm that the anti-HCC mechanisms of action of corylin are mediated by the upregulation of GAS5, we carried out a rescue assay. The results showed that corylin significantly inhibited the proliferation, migration, and invasiveness of HCC cells. By contrast, after silencing GAS5 expression, we found that the inhibitory effects of corylin on HCC cells were attenuated (Figure 6A–E), demonstrating that the anti-HCC mechanisms of corylin are mediated by its upregulation of GAS5.

## 3. Discussion

Corylin is a flavonoid component of *P. corylifolia* L. and, to the best of our knowledge, only occurs naturally in this plant. Although relevant studies have pointed out that corylin can promote bone differentiation and has anti-inflammatory properties [33,35,37], our understanding of the biological functions of corylin is currently limited. In addition, there are no studies examining its anticancer effects. In our study, we evaluated the anti-HCC activity of corylin. We found that corylin inhibited the proliferation, migration, and invasiveness of HCC cells and suppressed EMT by inducing the expression of lncRNA GAS5. The results of animal experiments also showed that corylin can inhibit the growth of tumor cells and the expression of EMT-associated proteins. To our knowledge, this is the first study to reveal that corylin can regulate lncRNAs to exert its antitumor effects.

To identify the genes and anticancer signaling pathways that may be regulated by corylin, we performed transcriptome profiling of corylin-treated cells. The results showed that corylin mainly regulates the expression of genes associated with the immune response, cell growth, and apoptosis; these data are consistent with the cell phenotype we observed. The findings suggest that corylin has the potential to become an adjuvant cancer treatment and may synergize with other anticancer drugs to increase their therapeutic efficacy.

Our study revealed that corylin induces lncRNA GAS5, which is a known anticancer lncRNA, and participates in the regulation of many important physiological processes, such as cell proliferation, apoptosis, the cell cycle, and EMT [44,47,48,49]. Some studies suggest that GAS5 is significantly downregulated in HCC tissues, and that GAS5 expression shows a positive correlation with patient prognosis [46,50]. In our study, we found that some of the inhibitory effects of corylin on HCC are due to GAS5-mediated anticancer mechanisms. Nevertheless, further studies are needed to understand how corylin upregulates GAS5 expression. In addition, our whole-transcriptome sequencing results showed that many lncRNAs are regulated by corylin, and the roles of these lncRNAs in the anticancer mechanisms of corylin require further research for elucidation.

The concentration of corylin in *P. corylifolia* L. is not high, and corylin has a simple structure. Thus, corylin in commercial formulations is mainly synthesized chemically. In the future, structural modifications may be introduced to increase its anticancer activity and absorption by cells. In our study, we employed cells and animal experiments to demonstrate that corylin has potential anticancer activity and affects some relevant regulatory mechanisms. As such, corylin may hold promise as a new therapeutic agent for HCC.

## 4. Materials and Methods 

### 4.1. Cell Lines, Antibodies, Drug, and siRNA

The HCC cell lines Huh7 and HepG2 were purchased from the American type culture collection (Manassas, VA, USA) and cultured in DMEM containing 10% fetal bovine serum at 37 °C in a 5% CO_2_ atmosphere. Polyclonal antibodies against *N*-cadherin, vimentin, slug, twist, snail, and β-actin were purchased from Genetex (Irvine, CA, USA) and Cell Signaling Technology (Beverly, MA, USA). The anti-mouse and anti-rabbit secondary antibodies were purchased from Santa Cruz Biotechnology (Santa Cruz, CA, USA). The corylin powder (purity above 98% as measured by HPLC) was purchased from Shanghai BS Bio-Tech Co., Ltd. (Shanghai, China) and dissolved in DMSO to a final concentration of 100 mM. Commercial si-GAS5 and negative-control siRNA were purchased from Thermo Fisher Scientific (Waltham, MA, USA).

### 4.2. Cell Proliferation Assay

Cell proliferation capacity was monitored with an xCELLigence real-time cell analyzer (ACEA Biosciences, Inc., San Diego, CA, USA) according to the manufacturer’s instructions. In brief, 3 × 10^3^ Huh7 or HepG2 cells were seeded into 96-well E-plates and maintained in DMEM containing different concentrations of corylin or vehicle only. The impedance value of each well was automatically monitored by the xCELLigence system for a duration of 72 h and expressed as a CI (cell index) value.

### 4.3. Cell Migration and Invasion Assays

The migration and invasion abilities of Huh7 and HepG2 cells were analyzed using wound-healing and Transwell migration assays as previously described [51]. 

### 4.4. Whole-Transcriptome Sequencing

Total RNAs were obtained from HCC cells treated with and without corylin using TRIzol reagent (Invitrogen, Carlsbad, CA, USA). The quality of RNA was checked by the Bioanalyzer 2100 system (Agilent Technologies, CA, USA). Ribosomal RNA (rRNA) was removed by the Epicentre Ribo-zero rRNA Removal Kit (Epicentre, Madison, WI, USA), and libraries were subsequently generated using the rRNA-depleted RNA and the NEBNext Ultra Directional RNA Library Prep Kit for Illumina (NEB, Ipswich, MA, USA) following the manufacturer’s instructions. The random hexamer primer and M-MuLV Reverse Transcriptase were used to generate first strand cDNA. DNA Polymerase I and RNase H were used to synthesize second strand cDNA. The approximately 150–200-bp cDNA fragments were purified with the AMPure XP system (Beckman Coulter, High Wycombe, Bucks, UK) after adenylation of the 3′ ends of the DNA fragments and ligation of the NEBNext Adaptor. Sequencing was performed using an Illumina Hiseq 2000 platform.

### 4.5. Detection of lncRNA GAS5 Levels Using Quantitative Real-Time RT-PCR

qRT-PCR was performed to determine the expression levels of lncRNA GAS5. Total RNA was obtained from cells using an RNeasy mini kit (QIAGEN, Gaithersburg, MD, USA), according to the manufacturer’s instructions. Two micrograms of RNA were reverse transcribed using the reverse transcription kit (Applied Biosystems, Foster City, CA, USA). These products were subjected to quantitative PCR to detect lncRNA GAS5 expression using the TaqMan gene expression assay (Applied Biosystems, Foster City, CA, USA); glyceraldehyde 3-phosphate dehydrogenase (GAPDH) was used as an internal control.

### 4.6. Transfection and Western Blotting

Huh7 and HepG2 cells were seeded into 6-well plates at a density of 3 × 10^5^ cells/well overnight. The cells were transfected with 50 µM siRNA (si-GAS5 or control siRNA (si-CTR)) using Lipofectamine RNAiMAX (Invitrogen, Carlsbad, CA, USA) according to the manufacturer’s instructions. Forty-eight hours after transfection, cells were washed twice with PBS and then lysed in 200 μL of RIPA lysis buffer (Thermo Fisher Scientific, MA, USA) containing protease inhibitors. Proteins (100 μg) were separated by electrophoresis with 12% SDS polyacrylamide gels, followed by Western blotting analysis to detect the levels of N-cadherin, vimentin, slug, twist, and β-actin. The immunoreactive bands were visualized using an enhanced chemiluminescence (ECL) kit (NEN Life Science Products, Boston, MA, USA) and developed using X-ray films. Each band was quantified using ImageQuant 5.2 (GE Healthcare, Piscataway, NJ, USA).

### 4.7. Xenograft Assays and Drug Administration

All experimental procedures involving animals were conducted in accordance with the Institutional Animal Care and Use Committee (IACUC) of Chang Gung Memorial Hospital. Six-week-old male nude mice (BALB/cAnN-Foxnlnu/CrlNarl) were purchased from the National Laboratory Animal Center (Taipei, Taiwan) and housed under pathogen-free conditions at the animal center of Chang Gung Memorial Hospital according to the Guidelines for the Care and Use of Laboratory Animals (NIH). Huh7 cells (5 × 10^6^) were subcutaneously implanted into the left and right flank regions of mice. All tumors were staged for one week before drug treatment was initiated. At the beginning of the second week, mice with tumors were intraperitoneally (IP) injected three times per week with 100 µL of corylin (at a dose of 60 mg/kg of body weight) or an equal volume of dimethyl sulfoxide (DMSO), which served as a control. Tumor volumes were measured three times per week using digital calipers. The mice were sacrificed 35 days after implantation. All the animal experiments were approved by the IACUC of Chang Gung Memorial Hospital (IACUC approval no. 2015121102, approval date: 1/26/2016).

### 4.8. Immunohistochemistry

Excised mouse tumors were fixed in formalin and embedded in paraffin. The 2-μm-thick consecutive sections were cut from the paraffin embedded tissue blocks and floated onto glass slides. Slides were first incubated at 65 °C for 1 h and then deparaffinized in xylene, rehydrated in graded ethanol solutions, and finally boiled in Trilogy reagent (Cell Marque, Rocklin, CA, USA) for 10 min for antigen retrieval. After washing with 1× PBS, the slides were immersed in 3% hydrogen peroxide for 10 min to suppress endogenous peroxidase activity. After triple-rinsing with 1× PBS, the sections were exposed to the appropriate primary antibodies for 1 h at room temperature, after which they were again triple-rinsed with 1× PBS and then incubated with a biotinylated secondary antibody (Dako, Glostrup, Denmark) for 25 min. After triple-rinsing with 1× PBS, the slides were treated with horseradish peroxidase-conjugated streptavidin for 25 min. The peroxidase activity was developed with 3,3′-diaminobenzidine (DAB, Dako), followed by counterstaining with hematoxylin.

### 4.9. Statistical Analysis

The original data of cell functional analyses, real-time PCR, and Western blotting were recorded as continuous variables and analyzed using Student’s *t*-test. All statistical analyses were performed using SPSS Statistical Software (Version 16, SPSS Inc., Chicago, IL, USA). All statistical tests were two-sided. The *p*-values of significance were established at <0.05 (*), <0.01 (**), or <0.001 (***).

## Abbreviations

HCC	Hepatocellular carcinoma
TCM	Traditional Chinese medicine
HPLC	High-performance liquid chromatography
CE	Capillary electrophoresis
GC	Gas chromatography
TLC	Thin-layer chromatography
lncRNA	Long noncoding RNA
EMT	Epithelial–mesenchymal transition
IC_50_	Half-maximal inhibitory concentration
DMSO	Dimethyl sulfoxide
